# Using Large Language Models to Predict Advanced Liver Fibrosis in Metabolic Dysfunction-Associated Steatotic Liver Disease (MASLD): A Proof-of-Concept Analysis

**DOI:** 10.7759/cureus.102777

**Published:** 2026-02-01

**Authors:** Basile Njei, Nelvis Njei, Sarpong Boateng, Omar Al Ta'ani, Yazan Al-Ajlouni

**Affiliations:** 1 Department of Medicine, Yale School of Medicine, New Haven, USA; 2 Department of Data Analysis, Centers for Machine Learning Intelligence (M-LINT), Ellicott City, USA; 3 Department of Medicine, Yale Affiliated Hospitals Program, Bridgeport, USA; 4 Department of Gastroenterology, Allegheny Health Network, Pittsburgh, USA; 5 Department of Rehabilitation, Montefiore Medical Center, New York, USA

**Keywords:** advanced liver fibrosis, gpt-4, large language models, metabolic dysfunction-associated steatotic liver disease (masld), risk prediction

## Abstract

Background: Metabolic dysfunction-associated steatotic liver disease (MASLD) is a prevalent condition linked to type 2 diabetes and other metabolic risk factors. Timely detection of advanced fibrosis (≥F3) in MASLD patients is critical for effective clinical management. Traditional risk scores, such as the Fibrosis-4 Index (FIB-4) and NAFLD Fibrosis Score (NFS), have limitations, prompting the exploration of machine learning models for improved risk prediction.

Objectives: This proof-of-concept study evaluates the feasibility of using large language models (LLMs), specifically GPT-4 and GPT-3.5 (OpenAI, Inc., San Francisco, United States), to predict advanced liver fibrosis in individuals with MASLD using only structured clinical variables from the National Health and Nutrition Examination Survey (NHANES).

Methods: We used NHANES 2017-2020 data, including 162 participants with MASLD. GPT-4 and GPT-3.5 were accessed via application programming interface (API) to predict fibrosis risk using variables such as age, BMI, aspartate aminotransferase (AST), alanine aminotransferase (ALT), platelet count, and HbA1c. Performance was evaluated using sensitivity, specificity, area under the receiver operating characteristic curve (AUROC), and Brier score, with model thresholds set at 40.5% for GPT-4 and 45% for GPT-3.5 based on Youden’s index.

Results: GPT-4 achieved an AUROC of 0.91 (95% CI: 0.86-0.96), while GPT-3.5 demonstrated an AUROC of 0.90 (95% CI: 0.85-0.95). Both models showed strong calibration, with GPT-4 maintaining superior specificity (0.86 vs. 0.82). The models' performance outpaced traditional risk scores, such as FIB-4.

Conclusions: GPT-based LLMs show strong potential for predicting advanced fibrosis in MASLD, offering a scalable, interpretable tool for clinical use. Further validation across diverse populations and clinical settings is needed to confirm generalizability and refine the approach before clinical adoption.

## Introduction

Metabolic dysfunction-associated steatotic liver disease (MASLD) represents a paradigm shift in the classification of liver disease, emphasizing the role of metabolic dysfunction rather than the exclusion of alcohol-related etiologies. Formerly termed non-alcoholic fatty liver disease (NAFLD), MASLD encompasses a continuum from simple steatosis to steatohepatitis, advanced fibrosis, cirrhosis, and hepatocellular carcinoma [1-3]. The condition is highly prevalent among individuals with type 2 diabetes and other metabolic risk factors, with estimates suggesting MASLD affects up to 67% of those with T2DM [4-6].

Timely identification of patients with advanced fibrosis (≥F3) is essential for guiding clinical management and preventing liver-related morbidity and mortality. Traditional risk scores such as Fibrosis-4 Index (FIB-4) and NAFLD Fibrosis Score (NFS) are limited by modest performance and frequently require follow-up imaging, constraining their use in primary care settings [7,8]. Meanwhile, new machine learning and AI-based approaches have shown promise in enhancing risk prediction and early detection. Interpretable models applied to structured data have demonstrated improved accuracy in identifying MASLD among high-risk populations, including hypertensive individuals and those with metabolic syndrome [9,10].

Large language models (LLMs), such as GPT-4 and GPT-3.5 (OpenAI, Inc., San Francisco, United States), represent a novel application of AI in clinical risk stratification [11]. These transformer-based models, traditionally used in natural language processing, are capable of synthesizing structured inputs into individualized, explainable risk predictions. Unlike traditional models, LLMs can dynamically adapt to new data and provide contextual rationales for predictions, offering a scalable tool for early fibrosis detection in routine care settings [12,13]. In this proof-of-concept study, we evaluate the feasibility of using GPT-based LLMs to predict advanced liver fibrosis in individuals with MASLD using only structured clinical variables. By leveraging publicly available NHANES data, we aim to assess whether transformer models can provide accurate, interpretable risk estimates based on routine laboratory and anthropometric features.

## Materials and methods

Study population

This study used publicly available data from the National Health and Nutrition Examination Survey (NHANES) 2017-2020, a program of the National Center for Health Statistics (NCHS), Centers for Disease Control and Prevention (CDC), Hyattsville, Maryland, United States. As this was a secondary data analysis of a national survey, no single hospital or institute was involved in data collection. The data analysis was performed at Yale School of Medicine, New Haven, Connecticut, United States. MASLD was defined in accordance with the 2023 MASLD consensus criteria: presence of hepatic steatosis (controlled attenuation parameter (CAP) ≥274 dB/m) and at least one cardiometabolic risk factor (CMRF), including BMI ≥ 25 kg/m² (23 kg/m² in Asians) or waist circumference (WC) >94 cm (males), 80 cm (females); fasting serum glucose ≥100 mg/dL or HbA1c ≥5.7% or history of type 2 diabetes or treatment for type 2 diabetes; blood pressure ≥130/85 mmHg or antihypertensive drug treatment; plasma triglycerides ≥150 mg/dL or lipid-lowering treatment; plasma high-density lipoprotein (HDL)-cholesterol ≤40 mg/dL (males), ≤50 mg/dL (females) or lipid-lowering treatment.

Exclusion criteria included viral hepatitis (positive hepatitis B core antibody or hepatitis C RNA), significant alcohol consumption (≥1 drink/day for women, ≥2 drinks/day for men), and other known causes of chronic liver disease where data were available, including autoimmune liver disease and congestive hepatopathy. Participants with clinical or laboratory features suggestive of alternative etiologies of cirrhosis were excluded when identifiable within NHANES. The race category "Others" includes Non-Hispanic Asian individuals, Non-Hispanic multiracial individuals, Other Hispanic individuals, and other races not classified in the primary NHANES categories. No protocol was pre-registered for this proof-of-concept study, and the study was not registered in a public registry or conducted with public involvement. 

Fibrosis classification

The fibrosis stage was estimated using a modified two-step approach aligned with the 2024 American Association for the Study of Liver Diseases (AASLD) guidance. Initially, participants were stratified using blood-based non-invasive liver disease assessment (NILDA) tools, FIB-4, and NFS into low, indeterminate, or high risk for advanced fibrosis. Subsequently, liver stiffness measurements (LSM) via vibration-controlled transient elastography (VCTE) were used to assign fibrosis stages (e.g., LSM 8-14 kPa for F3, ≥15 kPa for F4). A modified approach was used to assign patients to the higher end of overlapping categories (e.g., F2-F3 classified as F3).

The primary outcome of our study was advanced fibrosis (estimated fibrosis stage ≥3). Predictors included age, BMI, alanine aminotransferase (ALT), aspartate aminotransferase (AST), platelet count, estimated glomerular filtration rate (eGFR) calculated using the 2021 Chronic Kidney Disease Epidemiology Collaboration (GFR-EPI) equation, and hemoglobin A1c. These variables were selected based on prior evidence linking them to advanced liver fibrosis [14,15].

LLM prompt design

Two publicly available transformer-based LLMs developed by OpenAI’s GPT-4 and GPT-3.5 were accessed via application programming interface (API) through a secured Python interface (Python Software Foundation, Wilmington, Delaware, United States). A synthetic validation dataset of structured patient profiles was curated for this analysis, each consisting of the following seven variables: age (years), platelet count (×10³/µL), hemoglobin A1c (%), eGFR (mL/min/1.73 m²), BMI (kg/m²), AST (U/L), and ALT (U/L).

Each patient’s data was converted into a structured, human-readable text prompt to standardize the input format and minimize model variability. For example, a representative synthetic patient profile included the following values: age 63 years, platelet count 135 ×10³/µL, HbA1c 6.8%, eGFR 58 mL/min/1.73 m², BMI 28 kg/m², AST 35 U/L, and ALT 32 U/L. These variables were formatted into a single, human-readable prompt submitted to the model as follows: "You are a clinical decision support tool specializing in hepatology. You will receive structured patient data, including age, platelet count, HbA1c, eGFR, BMI, AST, and ALT. Based on these variables, estimate the probability (as a percentage) that the patient has advanced liver fibrosis (stage F3 or higher) in the context of metabolic dysfunction-associated steatotic liver disease (MASLD). Output your response in the following format:\nRisk percentage = [numerical value]%\nBrief reason: [concise clinical rationale for your prediction]\n\nBe precise, clinically grounded, and provide a percentage between 0 and 100."

The system instruction accompanying each batch of inputs directed the LLM to output both a numerical estimate of advanced fibrosis risk, phrased as "Risk percentage = [value]%", and a concise explanation. For the above example, the model returned: "Risk percentage = 55.9%\nBrief reason: Advanced age, borderline renal function, and impaired glucose control contribute to moderate fibrosis risk" [16].

**Figure 1:**
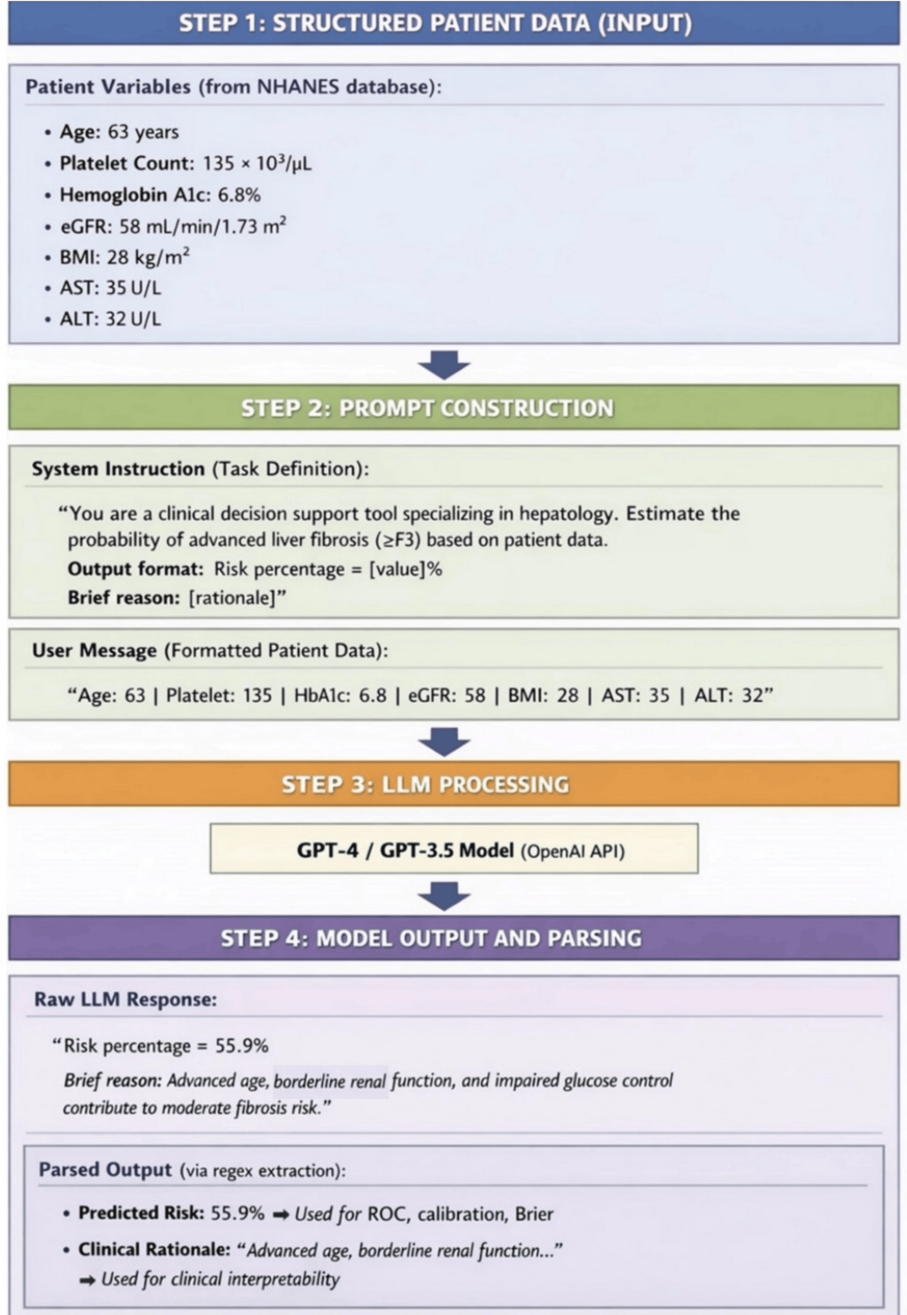
Workflow for converting structured patient variables into LLM prompts and parsed outputs LLM: large language model; NHANES: National Health and Nutrition Examination Survey; eGFR: estimated glomerular filtration rate; ALT: alanine aminotransferase; AST: aspartate aminotransferase; API: application programming interface

The numerical probability (0.559) was programmatically extracted using regular expressions and used for AUROC, calibration, and Brier score calculations. To ensure data quality, 100% of outputs were parsed successfully, and 20% of records were manually reviewed to verify correctness. The temperature was set to 0.0 for reproducibility, and identical synthetic validation cases were repeatedly tested across five runs, confirming consistent risk percentages. An overview of the entire process is illustrated in Figure 1. 

To optimize efficiency and reduce API call overhead, patient prompts were batched into groups of 20. Model responses were programmatically parsed to extract numerical values for predicted risk. If a batch failed to return valid responses due to API errors or timeouts, a retry mechanism was implemented to submit individual records within the batch.

Model performance evaluation

We applied Youden’s Index (J) to determine the optimal binary classification thresholds from the models' continuous risk outputs. This index, defined as J = Sensitivity + Specificity - 1, is a standard criterion for selecting a classification threshold that equally weights false positives and false negatives. As described in the Cochrane Handbook for Systematic Reviews of Diagnostic Test Accuracy, it provides a general index of test performance at a chosen cutoff, where values close to 1 indicate high accuracy and a value of zero indicates performance equivalent to random guessing. Youden’s Index identifies the point on the ROC curve that maximizes the sum of sensitivity and specificity. In our analysis, these optimal thresholds were 40.5% for GPT‑4 and 45% for GPT‑3.5. We evaluated model discrimination, calibration, and overall performance using the following metrics: sensitivity, specificity, area under the receiver operating characteristic curve (AUROC), F1 score, and the Brier score (a composite measure of calibration and accuracy).

We generated 95% confidence intervals (CIs) for each performance metric using bootstrap resampling with 1,000 iterations. Model calibration was assessed by plotting predicted vs. observed probabilities using calibration plots.

All analyses were conducted in Python (version 3.8) using the scikit-learn package for model evaluation and Seaborn/Matplotlib for visualization and graphical interpretation of results. A TRIPOD+AI statement, i.e., updated guidance for reporting clinical prediction models that use regression or machine learning methods, is provided in Appendix 1.

## Results

**Figure 2:**
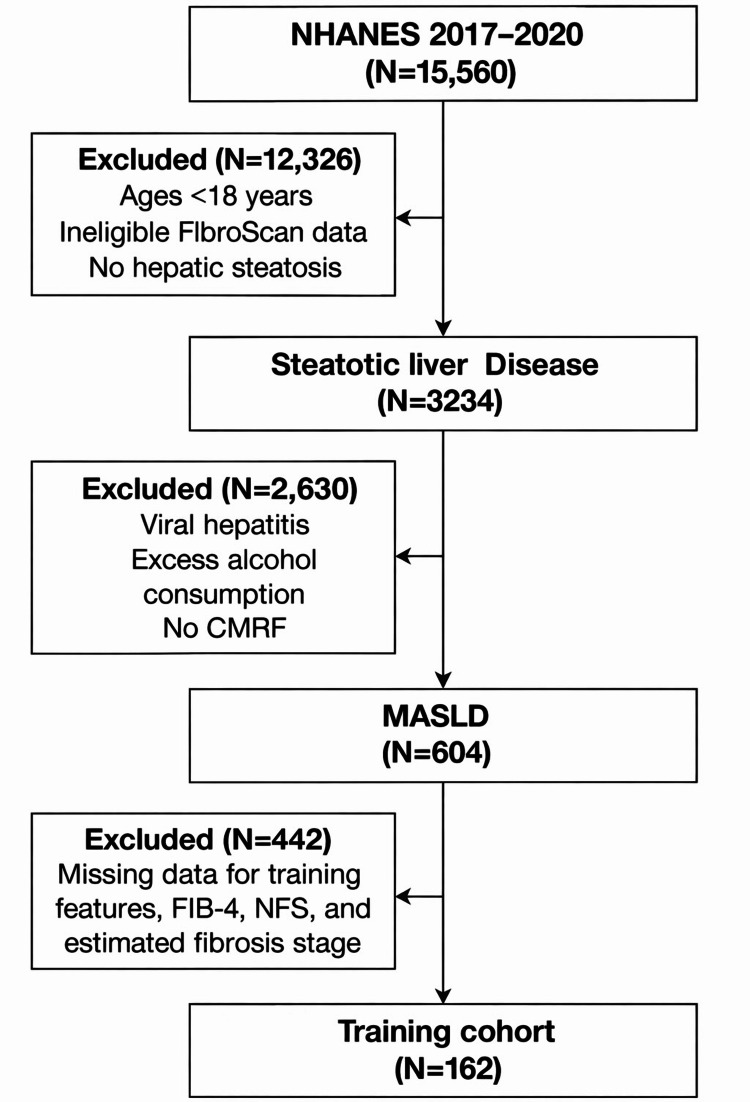
Flow chart NHANES: National Health and Nutrition Examination Survey; CMRF: cardiometabolic risk factor; MASLD: metabolic dysfunction-associated steatotic liver disease; NFS: NAFLD fibrosis score; FIB-4: Fibrosis-4 Index

A total of 15,560 participants from NHANES 2017-2020 were initially screened. Participants younger than 18 years, those without evidence of hepatic steatosis, or those without valid VCTE measurements were excluded, yielding 3,234 adults with steatotic liver disease. Among these, individuals with viral hepatitis, excess alcohol consumption, or without CMRFs were excluded, resulting in 604 participants meeting MASLD criteria. Of these, participants with missing data required for model inputs, fibrosis estimation, or comparator scores (FIB-4 and NFS) were excluded, leaving a final analytic cohort of 162 individuals for model development and evaluation (Figure 2).

Among the 162 MASLD participants, 84 (51.9%) were male, and the mean age was 49.5 (SD 16.3) years. Full cohort characteristics are shown in Table 1.

**Table 1:** Baseline characteristics of the derivation cohort (NHANES 2017-2020) CKD: chronic kidney disease; GFR: glomerular filtration rate; NAFLD: non-alcoholic fatty liver disease; National Health and Nutrition Examination Survey; HDL: high-density lipoproteins * denote variables that were included as input features in the model.

Variables	Training cohort (N=162)
Age*	49.5 (16.3)
Female	78 (48.1)
Male	84 (51.9)
Hispanic	53 (32.7)
Non-Hispanic Asian	18 (11.1)
Non-Hispanic Black	25 (15.4)
Non-Hispanic White	58 (35.8)
Other	8 (4.9)
Body-mass index (kg/m²)*	32.5 (5.8)
Alanine aminotransferase (U/L)*	24.4 (17.2)
Aspartate aminotransferase (U/L)*	21.6 (14.1)
Gamma glutamyl transferase (IU/L)	36.5 (48.6)
Platelet count (1000 cells/µL)*	256.8 (72.8)
Hemoglobin A1c (%)	6.0 (1.1)
Direct HDL-cholesterol (mg/dL)	47.8 (12.8)
Triglyceride (mg/dL)	156.5 (176.9)
2021 CKD-EPI GFR*	96.2 (20.5)
Creatinine (mg/dL)	0.8 (0.3)
Albumin (g/dL)	4.0 (0.3)
Obesity	157 (96.9)
Type 2 diabetes mellitus	124 (76.5)
Hypertension	105 (64.8)
Dyslipidemia	107 (66.0)
Fibrosis-4	1.0 (0.8)
NAFLD Fibrosis Score	0.3 (1.4)

**Figure 3:**
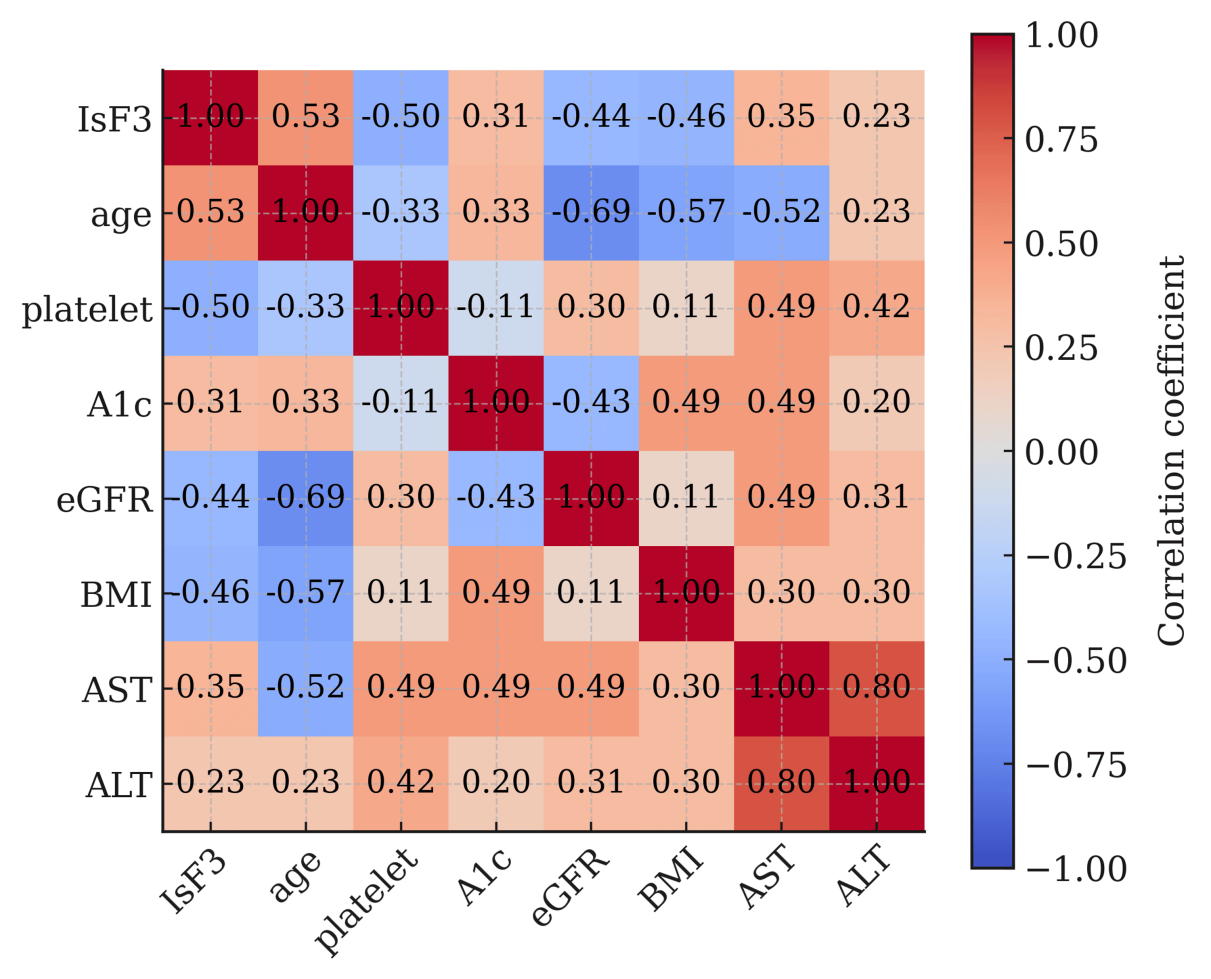
Correlation matrix eGFR: estimated glomerular filtration rate; ALT: alanine aminotransferase; AST: aspartate aminotransferase

Figure 3 presents the correlation matrix. AST and ALT were positively correlated (r = 0.80). Platelet count was inversely associated with fibrosis severity (r = -0.53). BMI showed a modest positive correlation with AST and ALT, suggesting metabolic risk interplay.

**Figure 4:**
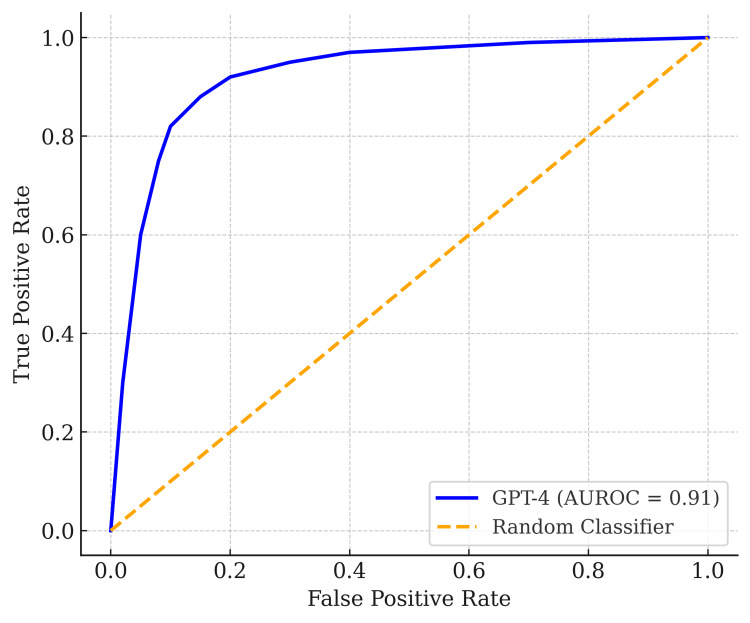
Receiver operating characteristic (ROC) curve for GPT-4, with an area under the receiver operating characteristic curve (AUROC) of 0.91 The diagonal orange line represents the random classifier reference.

Table 2 shows the performance metrics for GPT-4 and GPT-3.5, including 95% CIs via bootstrapping. GPT-4 displayed an AUROC of 0.91 (0.86-0.96), indicating excellent discriminative ability (Figure 4). Its Brier score was 0.12, signifying good calibration and overall accuracy. Meanwhile, GPT-3.5 showed comparable performance with an AUROC of 0.90 (0.85-0.95), a slightly lower Brier of 0.10, and higher sensitivity (0.91). GPT-4, however, maintained superior specificity (0.86 vs. 0.82).

**Table 2:** Performance metrics for GPT-4 and GPT-3.5 with 95% confidence intervals AUROC: area under the receiver operating characteristic curve

Model	AUROC (95% CI)	Brier (95% CI)	Sensitivity (95% CI)	Specificity (95% CI)	F1 Score (95% CI)
GPT-4	0.91 (0.86, 0.96)	0.12 (0.10, 0.14)	0.84 (0.71, 0.95)	0.86 (0.80, 0.92)	0.70 (0.57, 0.81)
GPT-3.5	0.90 (0.85, 0.95)	0.10 (0.08, 0.13)	0.91 (0.79, 1.00)	0.82 (0.76, 0.89)	0.69 (0.58, 0.80)

Table 3 illustrates examples of LLM-generated risk predictions with accompanying explanations. For instance, one patient’s prediction of 42.7% risk was accompanied by the explanation, 'Mildly elevated liver enzymes and borderline platelet count with metabolic risk factors (BMI, HbA1c).' These explanations help clinicians understand the rationale behind the model’s predictions, enhancing trust and supporting clinical validation.

**Table 3:** Example LLM predictions of advanced liver fibrosis risk in MASLD LLM: large language model; MASLD: metabolic dysfunction-associated steatotic liver disease

Patient Profile	LLM Risk Prediction	Model Explanation
Age: 50 | Platelet: 150 | HbA1c: 6.0 | eGFR: 85 | BMI: 30 | AST: 40 | ALT: 35	42.7%	Mildly elevated liver enzymes and borderline platelet count with metabolic risk factors (BMI, HbA1c).
Age: 60 | Platelet: 110 | HbA1c: 7.5 | eGFR: 65 | BMI: 33 | AST: 55 | ALT: 60	78.3%	Significantly elevated transaminases, thrombocytopenia, and diabetes suggest a high risk of advanced fibrosis.
Age: 45 | Platelet: 200 | HbA1c: 5.5 | eGFR: 95 | BMI: 24 | AST: 22 | ALT: 25	12.1%	Normal liver enzymes and platelets with a favorable metabolic profile suggest low risk of fibrosis.
Age: 72 | Platelet: 135 | HbA1c: 6.8 | eGFR: 58 | BMI: 28 | AST: 35 | ALT: 32	55.9%	Advanced age, borderline renal function, and impaired glucose control contribute to moderate fibrosis risk.
Age: 63 | Platelet: 175 | HbA1c: 6.3 | eGFR: 70 | BMI: 31 | AST: 38 | ALT: 40	47.5%	Mild liver enzyme elevation and insulin resistance indicate intermediate fibrosis risk.

**Figure 5:**
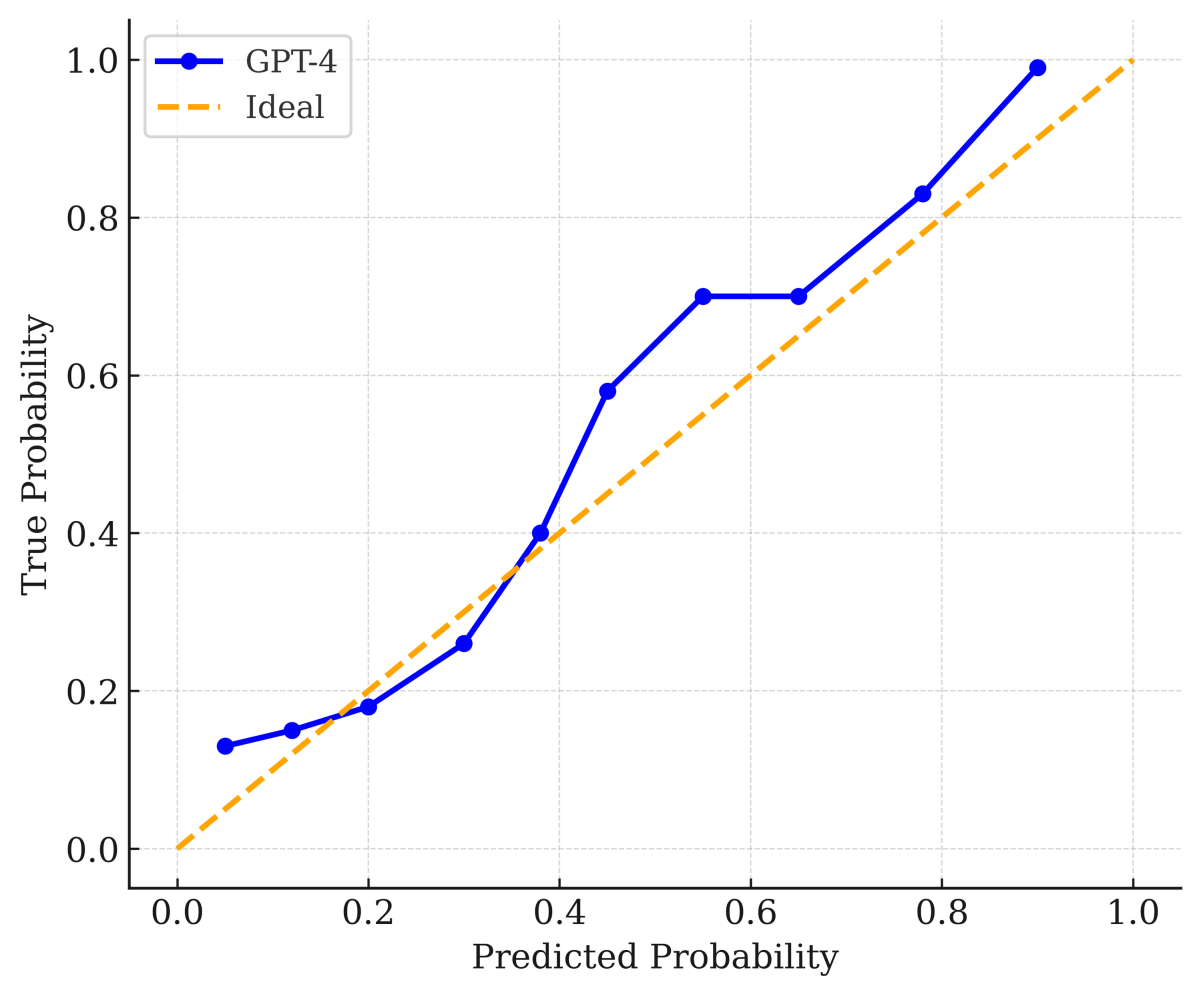
Calibration plot for GPT-4 Predicted probabilities (x-axis) align closely with observed frequencies (y-axis), suggesting good calibration. The blue line shows the relationship between predicted probabilities and observed event rates, while the orange dashed line represents perfect calibration (predicted probability = true probability).

Additionally, Figure 5 presents the calibration plot for GPT-4, where the predicted probabilities (x-axis) align closely with the observed outcomes (y-axis), suggesting good calibration. Calibration curves for both models indicated that predicted probabilities aligned well with observed risk. GPT-3.5’s calibration curve also approached the diagonal line, although there was minor underestimation at higher predicted probabilities.

## Discussion

This study aimed to evaluate the feasibility of using GPT-based LLMs to predict advanced fibrosis (≥F3) in individuals with MASLD using only structured clinical variables. By prompting GPT-4 and GPT-3.5 with basic anthropometric and laboratory data, we explored a novel, minimally invasive approach for fibrosis risk stratification that avoids the need for imaging or proprietary feature engineering [17]. Traditional non-invasive tests such as FIB-4 and NFS use fixed formulas based on a few laboratory parameters. In contrast, LLMs have the ability to recognize complex, non-linear patterns in clinical data that these conventional scores may miss.

This study is, to our knowledge, the first to apply general-purpose transformer-based LLMs for fibrosis prediction in MASLD, offering an interpretable, scalable, and real-time tool for frontline clinical deployment. The novelty of this approach lies in its dual advantages: leveraging off-the-shelf, publicly available LLMs with no task-specific fine-tuning; and enabling both numerical and narrative outputs to support clinician understanding and trust. In an environment where existing tools like FIB-4 and NFS are limited by either accuracy or clinical adoption, our model offers a streamlined alternative that may be easier to integrate into primary care settings [18]. For instance, Franck et al. demonstrated that FIB-4 underperformed in identifying significant fibrosis, with false-positive rates of 43% and false-negative rates of 26%, highlighting the risk of both over- and under-classification in routine practice [18]. The implications for early MASLD detection and triage are significant, especially in resource-limited contexts.

Overall, our findings demonstrated that both GPT-4 and GPT-3.5 performed well, with AUROCs of 0.91 and 0.90, respectively, values that exceed the typical range reported for traditional scores like FIB-4 (0.80-0.86) and NFS (0.73-0.86) in MASLD populations [19-21]. Prior research has highlighted FIB-4’s clinical simplicity and reasonable rule-out performance [22,23], but it still suffers from poor specificity and high indeterminate zones [24,25]. In our study, GPT-4 demonstrated higher specificity than GPT-3.5 (86% vs 82%), while GPT-3.5 yielded higher sensitivity (91% vs 84%), emphasizing the potential for tailored model selection depending on the desired trade-off between over- and under-referral.

These results compare favorably to machine learning models previously applied to MASLD and fibrosis prediction. For instance, Alizargar et al. reported strong performance (AUC 0.951) using XGBoost in gender-stratified NAFLD risk models, while Chang et al. employed deep learning on ultrasound data to achieve an AUC of 0.97 in fibrosis staging [26,27]. However, these models often depend on imaging features or proprietary feature sets, limiting their portability. Our approach required only seven routine variables, all available in standard electronic health record (EHR) systems, enabling frictionless integration into real-world clinical workflows.

While our previous work demonstrated that XGBoost-based models can accurately predict high-risk metabolic dysfunction-associated steatohepatitis (MASH) using routine clinical data, this study evaluates the transition to transformer-based LLMs. Unlike tree-based ensemble models, LLMs can flexibly scale to accommodate both structured and unstructured data, suggesting broader potential for future clinical applications [28]. 

In addition to the strong predictive performance demonstrated by both GPT-4 and GPT-3.5, an important feature of these models is their ability to provide human-readable rationales for each risk estimate. This transparency allows clinicians to identify the key variables driving a prediction, effectively refining real-time decision-making. This capability for generating rationale allows clinicians to validate the model’s outputs, ensuring that the decisions align with clinical knowledge and patient-specific factors. For instance, if the model indicates a high risk of advanced fibrosis due to elevated AST and ALT levels, specialists can focus on these markers when planning further diagnostic steps or therapeutic interventions. Furthermore, this interpretability fosters trust in AI-assisted decision-making, as healthcare providers are more likely to adopt tools they understand and can validate.

Clinical and public health implications

The application of GPT-based LLMs to fibrosis risk prediction has important implications for both clinical care and population-level screening strategies. Clinically, this low-cost approach enables rapid identification of high-risk individuals while providing interpretable rationales to support clinician decision-making and potentially reduce reliance on more invasive or expensive diagnostics such as liver biopsy or elastography, particularly in settings with limited access to hepatology expertise.

From a public health perspective, scalable tools that facilitate earlier identification of high-risk MASLD individuals could help shift liver disease management upstream, toward prevention and early intervention. This is especially relevant given the high and growing burden of MASLD. The use of LLMs could support opportunistic screening within primary care and community health settings, identifying candidates for lifestyle interventions, pharmacotherapy, or referral to specialty care before irreversible liver damage occurs. Moreover, the adaptability of LLMs enables real-time, scalable screening that aligns with evolving clinical guidelines to address gaps in MASLD detection.

Data privacy and cost-effectiveness considerations

While the clinical potential of GPT-based LLMs is promising, their integration into hepatology practice requires careful consideration of data protection and economic implications. LLMs, particularly when applied to patient-level data, raise concerns regarding the potential leakage of personal health information and the propagation of plausible but incorrect responses (error in interpretation) [29,30]. Future LLM deployment should prioritize privacy and security. In our study, models used only de-identified NHANES data, reducing immediate privacy risks. Real-world use will still require robust safeguards and transparency.

Strengths and limitations

This study has several notable strengths. First, it uses a publicly available, nationally representative data set (NHANES), increasing transparency and reproducibility. Second, it demonstrates that general-purpose LLMs can yield performance comparable to, or better than, specialized models without requiring retraining or proprietary infrastructure. Third, we enforced a standardized, human-readable input format, enabling reproducibility and interpretability at scale.

Limitations should also be considered. Our sample size was relatively small after applying strict inclusion criteria, and fibrosis was classified using a composite surrogate approach rather than histological staging. While practical, this may have introduced misclassification bias. The model’s performance has not yet been validated in external or prospective cohorts, and its generalizability beyond NHANES remains unknown. Additionally, GPT-generated explanations, while often clinically plausible, are not guaranteed to reflect causal relationships or pathophysiologic reasoning and should be used with appropriate caution. Although we excluded alternative etiologies of liver disease when identifiable, residual misclassification is possible given the survey-based nature of NHANES and the absence of histologic confirmation.

Importantly, GPT-4 and GPT-3.5 were used without task-specific fine-tuning, relying on public APIs. Fine-tuning with MASLD and AASLD guidelines could improve clinical precision and alignment with clinical standards, though further work is needed to validate this approach with real-world data and clinical inputs. Although the results are promising, further work is needed to validate this approach with real-world data and clinical inputs. 

## Conclusions

In this proof-of-concept study, GPT-4 and GPT-3.5 demonstrated excellent performance for advanced fibrosis risk prediction, with robust AUROC, acceptable calibration, and clinically relevant sensitivity and specificity tradeoffs. These findings highlight the potential for LLMs to enhance early detection of advanced fibrosis in patients with MASLD. However, the study is limited by the use of surrogate fibrosis measures, a relatively small sample size, and a lack of external validation. Future research should expand on these preliminary results to optimize and validate LLM-based screening in real-world clinical settings. With further validation, LLMs may complement traditional scoring systems and enable precision screening strategies in clinical hepatology.

## Appendices

Appendix 1 

**Table 4:** TRIPOD+AI statement: updated guidance for reporting clinical prediction models that use regression or machine learning methods

Section/Topic Item Development Checklist item / evaluation^1^				Reported on page
TITLE				
Title	1	D;E	Identify the study as developing or evaluating the performance of a multivariable prediction model, the target population, and the outcome to be predicted	1
ABSTRACT				
Abstract	2	D;E	See TRIPOD+AI for Abstracts checklist	2
INTRODUCTION				
Background	3a	D;E	Explain the healthcare context (including whether diagnostic or prognostic) and rationale for developing or evaluating the prediction model, including references to existing models	3-4
	3b	D;E	Describe the target population and the intended purpose of the prediction model in the context of the care pathway, including its intended users (e.g., healthcare professionals, patients, public)	3-4
	3c	D;E	Describe any known health inequalities between sociodemographic groups	NR
Objectives	4	D;E	Specify the study objectives, including whether the study describes the development or validation of a prediction model (or both)	4
METHODS				
Data	5a	D;E	Describe the sources of data separately for the development and evaluation datasets (e.g., randomised trial, cohort, routine care or registry data), the rationale for using these data, and representativeness of the data	5
	5b	D;E	Specify the dates of the collected participant data, including start and end of participant accrual; and, if applicable, end of follow-up	5
Participants	6a	D;E	Specify key elements of the study setting (e.g., primary care, secondary care, general population), including the number and location of centres	5, Table 1
	6b	D;E	Describe the eligibility criteria for study participants	5
	6c	D;E	Give details of any treatments received, and how they were handled during model development or evaluation, if relevant	N/A
Data preparation	7	D;E	Describe any data pre-processing and quality checking, including whether this was similar across relevant sociodemographic groups	5-6, Figure 1
Outcome	8a	D;E	Clearly define the outcome that is being predicted and the time horizon, including how and when assessed, the rationale for choosing this outcome, and whether the method of outcome assessment is consistent across sociodemographic groups	5
	8b	D;E	If outcome assessment requires subjective interpretation, describe the qualifications and demographic characteristics of the outcome assessors	N/A
	8c	D;E	Report any actions to blind assessment of the outcome to be predicted	N/A
Predictors	9a	D	Describe the choice of initial predictors (e.g., literature, previous models, all available predictors) and any pre-selection of predictors before model building	6
	9b	D;E	Clearly define all predictors, including how and when they were measured (and any actions to blind assessment of predictors for the outcome and other predictors)	6
	9c	D;E	If predictor measurement requires subjective interpretation, describe the qualifications and demographic characteristics of the predictor assessors	N/A
Sample size	10	D;E	Explain how the study size was arrived at (separately for development and evaluation), and justify that the study size was sufficient to answer the research question. Include details of any sample size calculation	7, Figure 1
Missing data	11	D;E	Describe how missing data were handled. Provide reasons for omitting any data	6
Analytical methods	12a	D	Describe how the data were used (e.g., for development and evaluation of model performance) in the analysis, including whether the data were partitioned, considering any sample size requirements	6
	12b	D	Depending on the type of model, describe how predictors were handled in the analyses (functional form, rescaling, transformation, or any standardisation).	6
	12c	D	Specify the type of model, rationale^2^, all model-building steps, including any hyperparameter tuning, and method for internal validation	6
	12d	D;E	Describe if and how any heterogeneity in estimates of model parameter values and model performance was handled and quantified across clusters (e.g., hospitals, countries). See TRIPOD-Cluster for additional considerations^3^	7
	12e	D;E	Specify all measures and plots used (and their rationale) to evaluate model performance (e.g., discrimination, calibration, clinical utility) and, if relevant, to compare multiple models	7-8
	12f	E	Describe any model updating (e.g., recalibration) arising from the model evaluation, either overall or for particular sociodemographic groups or settings	N/A
	12g	E	For model evaluation, describe how the model predictions were calculated (e.g., formula, code, object, application programming interface)	6
Class imbalance	13	D;E	If class imbalance methods were used, state why and how this was done, and any subsequent methods to recalibrate the model or the model predictions	NR
Fairness	14	D;E	Describe any approaches that were used to address model fairness and their rationale	11
Model output	15	D	Specify the output of the prediction model (e.g., probabilities, classification). Provide details and rationale for any classification and how the thresholds were identified	6, Table 3

Training versus evaluation	16	D;E	Identify any differences between the development and evaluation data in healthcare setting, eligibility criteria, outcome, and predictors	N/A
Ethical approval	17	D;E	Name the institutional research board or ethics committee that approved the study and describe the participant-informed consent or the ethics committee waiver of informed consent	14
OPEN SCIENCE				
Funding	18a	D;E	Give the source of funding and the role of the funders for the present study	14
Conflicts of interest	18b	D;E	Declare any conflicts of interest and financial disclosures for all authors	14
Protocol	18c	D;E	Indicate where the study protocol can be accessed or state that a protocol was not prepared	5
Registration	18d	D;E	Provide registration information for the study, including register name and registration number, or state that the study was not registered	5
Data sharing	18e	D;E	Provide details of the availability of the study data	14
Code sharing	18f	D;E	Provide details of the availability of the analytical code^4^	14
PATIENT & PUBLIC INVOLVEMENT				
Patient & Public Involvement	19	D;E	Provide details of any patient and public involvement during the design, conduct, reporting, interpretation, or dissemination of the study or state no involvement.	5
RESULTS				
Participants	20a	D;E	Describe the flow of participants through the study, including the number of participants with and without the outcome and, if applicable, a summary of the follow-up time. A diagram may be helpful.	5
	20b	D;E	Report the characteristics overall and, where applicable, for each data source or setting, including the key dates, key predictors (including demographics), treatments received, sample size, number of outcome events, follow-up time, and amount of missing data. A table may be helpful. Report any differences across key demographic groups.	5, Table 1
	20c	E	For model evaluation, show a comparison with the development data of the distribution of important predictors (demographics, predictors, and outcome).	N/A
Model development	21	D;E	Specify the number of participants and outcome events in each analysis (e.g., for model development, hyperparameter tuning, model evaluation)	5
Model specification	22	D	Provide details of the full prediction model (e.g., formula, code, object, application programming interface) to allow predictions in new individuals and to enable third-party evaluation and implementation, including any restrictions to access or re-use (e.g., freely available, proprietary)^5^	6
Model performance	23a	D;E	Report model performance estimates with confidence intervals, including for any key subgroups (e.g., sociodemographic). Consider plots to aid presentation.	7-8, Table 2
	23b	D;E	If examined, report results of any heterogeneity in model performance across clusters. See TRIPOD Cluster for additional details^3^.	7
Model updating	24	E	Report the results from any model updating, including the updated model and subsequent performance	N/A
DISCUSSION				
Interpretation	25	D;E	Give an overall interpretation of the main results, including issues of fairness in the context of the objectives and previous studies	9
Limitations	26	D;E	Discuss any limitations of the study (such as a non-representative sample, sample size, overfitting, missing data) and their effects on any biases, statistical uncertainty, and generalizability	11
Usability of the model in the context of current care	27a	D	Describe how poor quality or unavailable input data (e.g., predictor values) should be assessed and handled when implementing the prediction model	10
	27b	D	Specify whether users will be required to interact in the handling of the input data or use of the model, and what level of expertise is required of users	10
	27c	D;E	Discuss any next steps for future research, with a specific view to applicability and generalizability of the model	12
